# Immune Persistence Following a Single Dose of Varicella Vaccine: 5-Year and 8-Year Follow-Up of a Phase 3, Randomized, Double-Blind, Placebo-Controlled Trial

**DOI:** 10.3390/vaccines13101024

**Published:** 2025-09-22

**Authors:** Yanxia Wang, Xiangling Lei, Lili Huang, Yuehong Ma, Hongxue Yuan, Dongyang Zhao, Fanhong Meng

**Affiliations:** 1 Henan Centers for Disease Control and Prevention, Zhengzhou 450046, China; wangyanxia99@163.com (Y.W.); 13643826177@163.com (L.H.); 2 Sinovac Holding Group Co., Ltd., Beijing 100085, China; leixiangling9199@sinovac.com (X.L.); mayuehong8757@sinovac.com (Y.M.); yuanhx2052@sinovac.com (H.Y.); 3 Sinovac (Dalian) Vaccine Technology Co., Ltd., Dalian 116620, China

**Keywords:** immune persistence, varicella vaccine, children

## Abstract

**Background:** In recent years, breakthrough varicella cases among individuals who have received a single dose of varicella vaccine (VarV) have increased notably, suggesting that the long-term protection following a one-dose VarV regimen requires further investigation. This study aims to evaluate the immune persistence following a single dose of Sinovac VarV at 5 and 8 years post-vaccination. **Methods**: In this Phase 4, open-label, observational follow-up study, participants aged 1 to 12 years (referring to the age of vaccination) who had received a single dose of either Sinovac VarV or placebo in the previous phase 3 trial were enrolled in a 1:1 ratio. Blood samples were collected 5 years and 8 years post-vaccination to measure antibody levels against varicella using the fluorescent antibody to membrane antigen (FAMA) method. A total of 487 and 422 participants were included in the 5-year and 8-year immune persistence analyses, respectively. The endpoints comprised the seropositive rates (≥1:4 and ≥1:8) and the geometric mean titers (GMTs) of varicella antibodies at both 5 and 8 years following vaccination. **Results**: Varicella antibody levels declined from 30 days post vaccination but remained stable from 5 to 8 years. Five years after vaccination, the seropositive rates (≥1:4) of varicella antibody were 100% in the VarV group and 80.83% in the placebo group; for ≥1:8, the rates were 89.07% and 64.17%, respectively. The corresponding GMTs were 1:13.67 and 1:7.71, respectively. Eight years after vaccination, the seropositive rates (≥1:4) were 99.54% in the VarV group and 90.69% in the placebo group; for ≥1:8, they were 88.53% and 74.51%, with GMTs of 1:13.52 and 1:9.91, respectively. Eight years post-vaccination, the seropositive rates and antibody levels in the VarV group remained nearly the same as by 5 years. **Conclusions**: Sinovac VarV can confer good immune persistence for up to 8 years following a single dose of vaccination in children aged 1 to 12 years. However, given the declining trend in antibody levels over time, revaccination may be needed to maintain protective immunity.

## 1. Introduction

Varicella-zoster virus (VZV), also known as human herpesvirus 3, is a globally circulating alphaherpesvirus with a double-stranded DNA genome. Primary infection causes varicella (chickenpox) and establishes lifelong latency in sensory ganglia [1]. Clinical features of varicella commonly include a vesicular eruption with fever and malaise, which typically results in a mild to moderate illness but can also lead to severe complications, such as central nervous system involvement, pneumonia, secondary bacterial infections, and even death. Reactivation of VZV, which lies dormant in the spinal and cranial sensory ganglia after primary infection in childhood, can cause herpes zoster [2]. VZV is a highly contagious virus, with attack rates in susceptible contacts ranging from 61% to 100% [3]. It is predominantly a childhood illness, with over 90% of children in most countries testing seropositive by the age of 10 years [4,5].

Varicella continues to impose a substantial public-health burden. The World Health Organization estimates that about 140 million varicella cases occur globally each year, leading to 4.2 million severe complications requiring hospitalization and approximately 4200 deaths [6]. Since the development of the first live attenuated varicella vaccine (VarV) in 1974 [7], VarV has been widely recognized as the most practical and effective strategy for the prevention and control of varicella [8,9]. In China, VarV became available in 1998, but is not included in the National Immunization Program, and most areas use a self-paid, mainly one-dose schedule; first-dose coverage is ~80–93% while second-dose coverage is only ~48.7–72.9% [10]. Correspondingly, incidence remains high, especially in children: in 2021 the highest rate was in ages 5–9 years (~224 per 100,000), with adolescents 15–19 years (180/100,000) exceeding 10–14 years (123/100,000) and 0–4 years (106/100,000) [11]. By contrast, the United States has a routine two-dose schedule (ACIP), following licensure in 1995 and a shift from one-dose (1995–2005) to two-dose (since 2006); by 2018–2019 overall varicella incidence fell to under 10/100,000 in children [12], with 92–95% declines among children 5–14 years [10]. Germany introduced universal childhood varicella vaccination in 2004 and later adopted a two-dose program; by the 2020 school-entry exam, coverage reached ~88.9% for dose 1 and 85.1% for dose 2, and incidence in children <9 years dropped from >3000/100,000 in 2006 to 40–85/100,000 in 2022.

The vaccine effectiveness (VE) for a single dose has been reported to range from 72.9% to 95.8% [13,14,15]. However, the VE of one-dose VarV vaccination waned over time [14,15,16]. Some studies in China demonstrated that the peak of breakthrough incidence occurred about 3–7 years after one-dose VarV vaccination [17,18]. In recent years, varicella outbreaks increased notably, accompanied by a rising proportion of breakthrough cases occurring in individuals with a documented history of one-dose VarV [18,19]. Most breakthrough cases occurred in primary and secondary school students [17,18,20]. These observations have raised questions regarding the immune persistence following a single dose of VarV in children. Some studies have demonstrated that varicella antibodies tend to decline over time, with a seropositivity rate of only 48.28% three years after vaccination [21]. In contrast, another study reported that the seropositivity rate remained above 90% in healthy children 11 years after receiving a single dose of VarV [22]. Therefore, studies into the long-term immune persistence following a one-dose VarV regimen is still warranted.

In 2016, a Phase 3 clinical trial was conducted for a VarV developed by Sinovac (Dalian) Vaccine Technology Co., Ltd., Liaoning, China (Sinovac VarV), which demonstrated satisfactory vaccine efficacy and safety in children aged 1 to 12 years [23]. Here, we present the results of the follow-up phase 4 study based on data collected 5 and 8 years after vaccination, and provide additional evidence on the persistence of immunity induced by a single dose of Sinovac VarV.

## 2. Materials and Methods

### 2.1. Study Design

This Phase 4, open-label, observational follow-up study was built based on the previous Phase 3 clinical trial (ClinicalTrials.gov Identifier: NCT02981836) [23], which is a double-blind, randomized, placebo-controlled study in healthy children aged 1 to 12 years in China. In this Phase 3 clinical trial, a total of 5997 children were enrolled and randomly assigned to receive either Sinovac VarV or placebo in a 1:1 ratio. Among them, 703 participants (349 from the VarV group and 354 from the placebo group) were included in the immunogenicity per-protocol set (PPS).

This phase 4 follow-up study aimed to enroll at least 480 participants who had been included in the immunogenicity PPS of the parent Phase 3 study, with 240 participants in the VarV or placebo groups, respectively. The phase 4 study was conducted in Xiangcheng and Biyang counties of Henan province, China from December 2021 to February 2025. All participants and/or their legal guardians provided written informed consent at enrollment. This study was approved by the Institutional Review Board of Henan Provincial Centers for Disease Control and Prevention (approval number: 2021-YM-017-02), and was conducted in accordance with the Declaration of Helsinki, good clinical practice (GCP), and all applicable ethical standards. The study is registered at ClinicalTrials.gov with the identifier NCT05095701.

### 2.2. Participants

In this phase 4 study, participants who had valid varicella antibody titer measurements both before and 30 days after VarV vaccination in the parent phase 3 study were enrolled. Participants were excluded if they met any of the following exclusion criteria prior to blood collection 5 and 8 years post-vaccination, respectively: (a) had a documented history of varicella or herpes zoster; (b) had a documented history of varicella vaccination after the phase 3 clinical study; (c) were diagnosed with autoimmune diseases, immunodeficiency, or were undergoing immunosuppression; (d) had a documented history of immunosuppressant treatment after the phase 3 clinical study.

### 2.3. Vaccines

In the parent phase 3 study, participants received a single dose of Sinovac VarV or placebo. The live attenuated VarV was derived from the Oka strain of the attenuated varicella–zoster virus and was propagated in human diploid SV-1 cells. The potency of the VarV was not less than 3.3 lgPFU per dose (0.5 mL). The placebo was prepared using the same methodology as the VarV and contained the same excipients and diluent as the vaccine but no viral antigen. During the current phase 4 study, no additional doses were administered to participants.

### 2.4. Immunogenicity Assessment

Blood samples of approximately 3.0 to 3.5 mL were collected from each participant 5 years and 8 years post-vaccination, respectively, to assess immune persistence. The blood collection period allowed for a six-month time window on each occasion. Blood was processed within 2 h; serum stored at −20 °C and batch-tested at each period’s end. Five- and eight-year samples were assayed in separate batches in the same lab using the same fluorescent antibody to membrane antigen (FAMA) protocol.

For the FAMA test, fluorescent assay plates were prepared with suspensions of Oka-strain VZV in diploid cells and stored at −70 °C. Sera were isolated, inactivated in a 56 °C water bath for 30 min, then stored at −20 °C. Two-fold serial dilutions were dispensed into wells alongside the control samples. Following incubation with detection antibodies and Evans blue, results were read under a fluorescence microscope. Procedures were fully documented, and laboratory staff were blinded to allocation.

In line with other one-dose investigations and the parent phase 3 trial VZV3001 [23], a serum titer of ≥1:4 is generally considered seropositive. The Phase 3 study further evaluated a surrogate immunologic marker and indicated that a FAMA titer ≥1:8 may confer protection. Therefore, both ≥1:4 and ≥1:8 cut-offs were used in later phase 4 research. The seropositive rate (≥1:4) was defined as the proportion of participants with a varicella antibody titer of at least 1:4, while the seropositive rate (≥1:8) referred to the proportion of participants with a titer of at least 1:8. The endpoints comprised the seropositive rates (≥1:4 and ≥1:8) and the geometric mean titers (GMTs) of varicella antibodies at both 5 and 8 years following vaccination.

### 2.5. Sample Size Determination and Statistical Analysis

Based on prior epidemiological data and clinical study results [24,25], it was estimated that, 5 years after vaccination, the seropositive rate of varicella antibodies (≥1:8) would be 50% in the placebo group and 80% in the VarV group. With a significance level of α = 0.05 (two-sided) and a statistical power of 90%, the minimum sample size required was 51 participants per group. Similarly, 8 years after vaccination, the seropositive rate of varicella antibodies (≥1:8) was projected to be 60% in the placebo group and 75% in the VarV group, resulting in a minimum sample size requirement of 203 participants per group.

To ensure adequate statistical power, the larger of the two calculated sample sizes was selected, requiring a minimum of 203 cases per group. To account for potential variations arising from invalid serum samples and uncertainties in antibody seropositive rate estimates, an additional 15% was added to the initial sample size. Consequently, the final sample size was set at approximately 240 participants per group, resulting in a total sample size of 480 participants.

The 5-year immune persistence set (IPS) comprises individuals who provided blood samples and had valid varicella antibody titer measurements available five years after vaccination. The 8-year immune persistence set (IPS2) consists of participants from the IPS who completed an additional blood collection eight years post vaccination, with valid varicella antibody titer measurements also obtained.

The statistical analyses were conducted using SAS 9.4 software (SAS Institute Inc., Cary, NC, USA). The seropositive rate of varicella antibodies was estimated, and the corresponding 95% confidence interval (CI) was calculated using the Clopper–Pearson method. Comparisons between groups were performed using either the chi-square test or Fisher’s exact test, as appropriate. The GMT was calculated based on the geometric mean and the associated 95% confidence interval. Differences between groups were further assessed using paired t-tests after logarithmic transformation of the data.

## 3. Results

### 3.1. Study Participants

**Figure 1:**
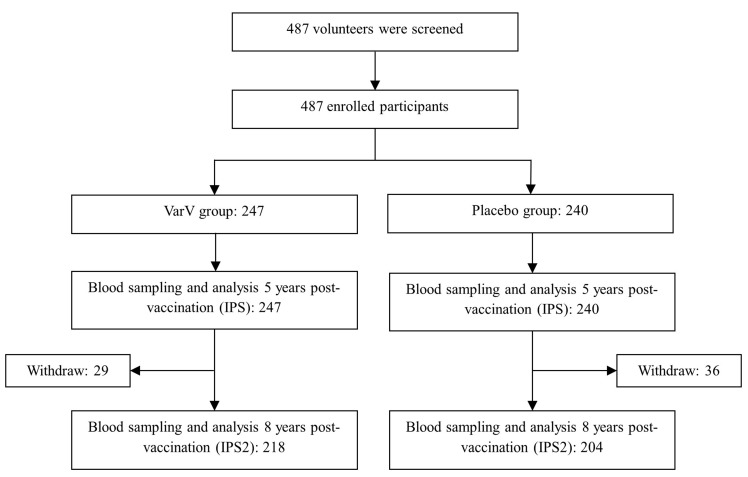
Flowchart of the Phase 4 Clinical Study and Participant Analysis Dataset. Abbreviations: VarV, varicella vaccine; IPS, 5-year immune persistence set; IPS2, 8-year immune persistence set.

A total of 487 participants were enrolled in this study, comprising 247 in the VarV group and 240 in the placebo group. All 487 participants had valid varicella antibody titer values 5 years post vaccination and were included in IPS, of which 422 were included in IPS2 (Figure 1 and Table S1).

The mean age at vaccination was 6.3 years in IPS and 6.1 years in IPS2, and 49.9% of participants in IPS and 49.3% in IPS2 were female, respectively. No statistically significant differences in age and gender were observed between groups in both IPS and IPS2, suggesting that the demographics characteristics of participants between two groups were balanced and comparable (Table 1). 

### 3.2. Immunogenicity and Immune Persistence in Overall Population

#### 3.2.1. 5-Year Immune Persistence Analysis

**Figure 2:**
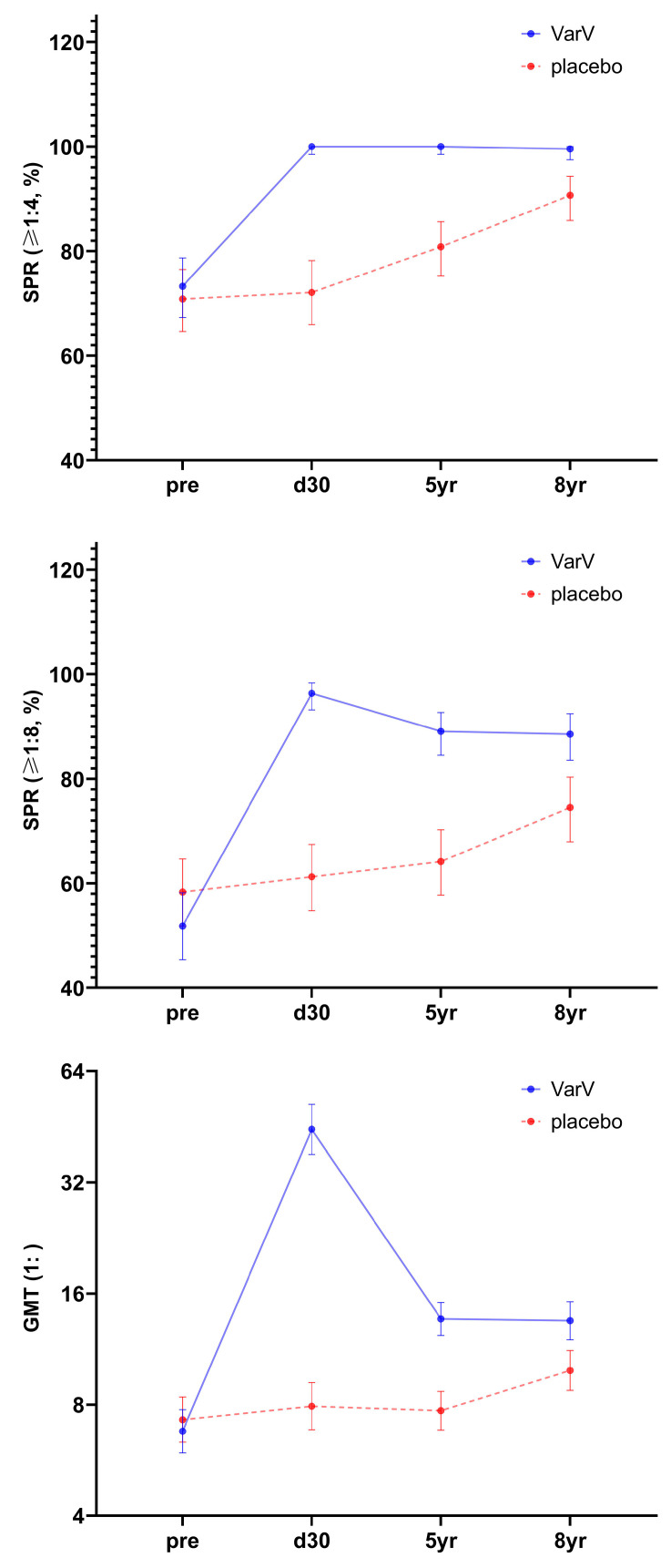
Seropositive rates and GMTs at different time points before and after VarV vaccination (IPS2). Abbreviations: VarV, varicella vaccine; SPR, seropositive rate; GMT, geometric mean titer; IPS2, 8-year immune persistence set.

Five years after vaccination, the seropositive rates for varicella antibodies (≥1:4) were 100% in the VarV group and 80.83% in the placebo group, while the seropositive rates (≥1:8) were 89.07% and 64.17%, respectively. The corresponding GMTs were 1:13.67 in the VarV group and 1:7.71 in the placebo group (Figure 2). Notably, the VarV group demonstrated significantly higher seropositive rates at both criteria (≥1:4 and ≥1:8) as well as higher GMTs compared to the placebo group.

#### 3.2.2. 8-Year Immune Persistence Analysis

Eight years after vaccination, the seropositive rates of varicella antibody (≥1:4) in the VarV group and the placebo group were 99.54% and 90.69%, while the seropositive rates (≥1:8) were 88.53% and 74.51%, respectively. The corresponding GMTs were 1:13.52 in the VarV group and 1:9.91 in the placebo group (Figure 2). Both the seropositive rates at predefined titer criteria and the GMT values remained significantly higher in the VarV group than in the placebo group.

#### 3.2.3. Full Series Immune Persistence Analysis

Before the vaccination, no statistically significant differences were observed in the seropositive rates (both ≥1:4 and ≥1:8) or GMTs between the two groups. From one month post vaccination to 8 years post-vaccination, at various observation time points, the seropositive rates (≥1:8) and GMT of varicella antibody in the VarV group were significantly higher than those in the placebo group. The results of IPS2 at 8 years post-vaccination were essentially consistent with those of IPS at 5 years post-vaccination.

Compared to 30 days post-vaccination, antibody levels significantly declined at 5 and 8 years after a single dose of VarV vaccination; however, they remained higher than pre-vaccination levels. Additionally, compared with the 5-year time point, the seropositive rates for varicella antibodies (≥1:4 and ≥1:8) as well as GMTs in the VarV group remained largely stable at 8 years post-vaccination (Figure 2 and Table S2).

### 3.3. Immunogenicity and Immune Persistence in Different Age Subgroups

Five years post-vaccination, seropositive rates (≥1:4) were 100% in all age subgroups in the VarV group, compared to 77.78%, 82.54%, and 80.39% in the placebo group for the 1–4 years, 5–8 years, and 9–12 years age subgroups, respectively. For seropositive rates (≥1:8), the VarV group showed rates of 88.24%, 87.94%, and 92.73% across the three age subgroups, whereas the placebo group showed 60.32%, 65.08%, and 66.67%, respectively. The GMTs were 10.79, 13.60, and 17.26 in the VarV group, and 6.93, 7.61, and 9.04 in the placebo group for the 1–4 years, 5–8 years, and 9–12 years age subgroups, respectively.

Eight years post-vaccination, seropositive rates (≥1:4) were 100%, 99.22%, and 100% in the VarV group, and 86.67%, 92.45%, and 92.11% in the placebo group for the 1–4 years, 5–8 years, and 9–12 years age subgroups, respectively. Seropositive rates (≥1:8) were 87.23%, 86.72%, and 95.35% in the VarV group, and 63.33%, 76.42%, and 86.84% in the placebo group for the same age subgroups. The GMTs were 11.74, 13.17, and 17.07 in the VarV group, and 8.48, 9.80, and 13.09 in the placebo group for the 1–4 years, 5–8 years, and 9–12 years age subgroups, respectively.

Five and eight years after vaccination, the GMTs and seropositive rates in the VarV groups of all age strata decreased significantly compared to 30 days post-vaccination. However, they remained higher than the pre-vaccination levels and those of the placebo groups. Except for the GMT in the 1–4 years age stratum and the seropositive rates (≥1:4 and ≥1:8), as well as the GMT in the 9–12 years age stratum eight years after vaccination, at various observation time points, the antibody levels and seropositive rates in the VarV group were higher than those in the placebo group, with statistically significant differences.

The changes in antibody levels in different age subgroups from the baseline before the vaccination in Phase 3 to 8 years after the vaccination are shown in Table 2.

## 4. Discussion

One dose of VarV was moderately effective in preventing all varicella and highly effective in preventing moderate/severe varicella cases [26,27]. However, this regimen does not effectively interrupt circulation among school-aged children [18,27]. Several studies suggest that the incidence of breakthrough varicella cases among children who have received a single dose of VarV has been increasing, with up to 93.38% of breakthrough cases having a history of single-dose VarV vaccination [18,19]. The peak incidence of breakthrough varicella cases occurs in 3 to 7 years after vaccination [18]. This increasing risk implies that the long-term effectiveness of a single dose of VarV needs to be evaluated, and an additional vaccination may be required. In China, follow-up data on immune persistence beyond 5 years after vaccination remain limited. This study provides comprehensive data on the 5- and 8-year immune persistence of VarV among participants who received one dose of Sinovac VarV at the age of 1 to 12 years during the pivotal phase 3 clinical trial.

In the present study, we demonstrated that the immunogenicity against varicella remained at a high level at both 5th and 8th years post vaccination, although antibody levels had significantly declined from 30 days post-vaccination to 5 years. These findings suggest that a single dose of the VarV can induce long-lasting immunity, characterized by an initial decline in antibody levels followed by stabilization.

Results of this study are largely consistent with the previous findings [22,28,29,30,31]. The antibody levels observed 5 years post vaccination in this study are largely consistent with those reported in other studies [28,29,30]. According to a study by Huang et al. [22], varicella antibody GMTs, as measured by the FAMA method, decreased approximately fourfold in children aged 1–12 years 11 years after receiving a single dose of VarV vaccine compared to levels measured 30 days post-vaccination. This decline is more pronounced than what was observed in this study at both 5 years and 8 years post-vaccination, where the reduction was approximately 3.3-fold. Another study [28] reports that the seropositive rate of varicella antibodies (≥1:4) among 6 to 7 years old children 5 years after one-dose vaccination was 89.05%, with a GMT of 1:8.21, which values slightly lower than those found in this study. Compared to 3 months post-primary vaccination, varicella antibody levels declined by approximately 3.7-fold 5 years later, again indicating a more pronounced decrease than in this study. Another study by Su et al. [29] indicates that among children aged 1 to 6 years, 5 years after single-dose vaccination of VarV, the test group had a varicella antibody seropositive rate (≥1:4) of 98.76% (GMT: 1:23.62), while the positive control group had a seropositive rate (≥1:4) of 94.59% (GMT: 1:10.59). The seropositive rates of both groups were comparable to those in this study. However, the GMT in the VarV group was higher, and that in the seropositive control group was slightly lower than that in this study. A separate cross-sectional survey [30] revealed that among 566 children aged 2 to 7 years who had received a single dose VarV, the antibody seropositive rate (≥1:4) was 93.7% with a GMT of 1:11.51 one year post vaccination, and 89.9% with a GMT of 1:8.36 five years post-vaccination. Both values were slightly lower than those observed in this study. The discrepancies in immune persistence data across studies may be partially attributed to variations in sample sizes and differences in exogenous exposure risks among study regions. Overall, the immune persistence of the VarV observed in this study was comparable to that reported in other studies.

A placebo-controlled design was utilized to evaluate the impact of natural infection on varicella antibody levels in this study, thereby enabling a precise assessment of the immune persistence of the VarV vaccine. Because both arms of the parent trial were followed under the same community conditions, the placebo arm serves as a contemporaneous benchmark of exogenous (background) exposure to varicella. Over the 5- and 8-year follow-up we observed marked increases in seropositivity and GMTs in placebo recipients, despite excluding participants with documented varicella or herpes zoster and censoring incident cases. This pattern mirrors long-term cohorts in which unvaccinated children progressively seroconvert through repeated exposure and asymptomatic infection, and underscores that the high seropositivity observed in our placebo group reflects genuine exogenous boosting rather than assay noise or cross-reactivity [32,33]. Our estimates therefore reflect immune persistence under real-world endemic exposure rather than a hypothetical no-exposure counterfactual. Although both groups shared the same exposure environment—which can elevate varicella antibody levels independent of vaccination—vaccine recipients consistently exhibited higher seropositivity and GMTs than placebo recipients, indicating durable vaccine-induced immunity beyond what natural exposure alone affords.

Though the antibody levels observed 8 years after vaccination in this study were lower than those measured 30 days post-vaccination, a certain level of protective immunity may still be maintained. A study conducted in Japan [31] demonstrated that among healthy children who had received VarV 7 to 10 years prior and had close contact with varicella patients, 95% (101/106) did not develop the disease. Among the 38 children for whom serological results were available (measured by FAMA), 97% (37/38) tested positive for varicella antibodies, with a GMT of 1:9.3. 

However, this strategy was insufficient to interrupt varicella transmission among school-age children [18,27]. Despite high seropositive proportions 5 and 8 years post one-dose vaccination, GMTs declined over time, and breakthroughs could happen subsequently. Several studies suggest that the incidence of breakthrough varicella cases among children who have received a single dose of VarV has been increasing, with up to 93.38% of breakthrough cases having a history of single-dose VarV vaccination [18,19]. Furthermore, epidemiological investigations have shown that in regions of China with high coverage of a single dose of VarV, varicella outbreaks continue to occur periodically, primarily within primary and secondary schools [34,35]. According to the WHO position paper on varicella and herpes zoster vaccines [6], a single dose can significantly reduce both the incidence and mortality associated with varicella; however, it is insufficient to prevent localized viral transmission and outbreaks. Vaccination of a second dose has been shown to further decrease the number of cases and the frequency of outbreaks. Compared to the United States and Germany, which adopted the WHO recommended two-dose program, varicella incidence among children in China is still at a relatively high level [10,11,12]. Based on the epidemiological data on varicella and the findings of this study, it may be necessary to receive a booster dose at an appropriate age.

We observed that age may have a minor influence on the response to vaccination. Similar immune responses were detected across the 1–4 years, 5–8 years, and 9–12 years age subgroups at 30 days following a single dose of VarV vaccination. Furthermore, the antibody level changes exhibited a consistent trend across all age groups throughout the 8-year follow-up period. In the 1–4 years old and 9–12 years old subgroups, certain immunogenicity differences between the groups were no longer statistically significant, although the values in the VarV group remained higher than those in the placebo group. This may be attributed to the relatively small sample sizes within these subgroups. Our results suggest that a single dose of VarV may confer protection against varicella, with immunity persisting for at least 5 to 8 years following vaccination in children aged 1 to 12 years at the time of vaccination. These individuals may reach the peak incidence age range [11,18] within the 8-year follow-up period. Nevertheless, further studies are needed to evaluate the long-term immune response to VarV in other age groups and in larger populations.

One limitation of this study is that, although a placebo-controlled design was employed to minimize the influence of exogenous exposure on varicella antibody measurements, the generalization of these findings to other regions may be constrained by regional differences in varicella prevalence. Furthermore, the objective of this follow-up was to assess the persistence of vaccine-induced immunity, quantified by seropositivity and GMTs at years 5 and 8. We therefore excluded participants with documented varicella or zoster to avoid conflating vaccine persistence with infection-induced boosting, which would artificially inflate seropositivity and GMTs and overestimate persistence. As a result, vaccine effectiveness or breakthrough cases due to vaccine failures could not be evaluated in this study, highlighting the need for further research.

## 5. Conclusions

In conclusion, 5 years and 8 years after receiving a single dose of Sinovac VarV, children aged 1 to 12 years maintained a relatively high varicella antibody seropositivity rate. Although antibody levels exhibited a declining trend over time, they remained significantly higher than those observed in the placebo group, suggesting that the vaccine induces long-lasting immunogenicity. To further reduce the risk of varicella breakthrough infection, the timely vaccination of a booster dose may be warranted.

## Figures and Tables

**Table 1:** Participant demographics and other baseline information.

Analysis Dataset	Variable	VarV Group (N = 247)	Placebo Group (N = 240)	Total (N = 487)	*p*-Value
IPS	N	247	240	487	
	Age of vaccination (years, mean ± SD)	6.5 ± 2.8	6.2 ± 2.8	6.3 ± 2.8	0.2898
	Gender (male, %)	51.4%	48.8%	50.1%	0.5562
IPS2	N	218	204	422	
	Age of vaccination (years, mean ± SD)	6.3 ± 2.7	6.0 ± 2.8	6.1 ± 2.7	0.2185
	Gender (male, %)	51.8%	49.5%	50.7%	0.6331

Abbreviations: IPS, 5-year immune persistence set; IPS2, 8-year immune persistence set; SD, standard deviation; VarV, varicella vaccine.

**Table 2:** The 5-year and 8-year immunological persistence of VarV after vaccination in different age groups (age of vaccination).

Time Point	Variable	1–4 Years Old			5–8 Years Old			9–12 Years Old		
		VarV Group	Placebo Group	*p*-Value	VarV Group	Placebo Group	*p*-Value	VarV Group	Placebo Group	*p*-Value
**Before vaccination**	N	51	63		141	126		55	51	
	Seropositive (≧1:4) rate % (95%CI)	39.22 (25.84, 53.89)	47.62 (34.88, 60.59)	0.3686	79.43 (71.82, 85.77)	74.60 (66.08, 81.93)	0.3481	89.09 (77.75, 95.89)	90.20 (78.59, 96.74)	0.8521
	Seropositive (≧1:8) rate % (95%CI)	29.41 (17.49, 43.83)	38.10 (26.15, 51.20)	0.3312	53.90 (45.31, 62.32)	57.94 (48.82, 66.67)	0.5074	67.27 (53.29, 79.32)	84.31 (71.41, 92.98)	0.0416
	GMT (1:) (95%CI)	3.44 (2.79, 4.25)	4.23 (3.36, 5.31)	0.1998	7.50 (6.25, 9.01)	7.21 (5.98, 8.68)	0.7586	9.79 (7.43, 12.90)	14.75 (10.78, 20.18)	0.0508
**30 days after vaccination**	Seropositive (≧1:4) rate % (95%CI)	100.00 (93.02, 100.00)	52.38 (39.41, 65.12)	<0.0001	100.00 (97.42, 100.00)	74.60 (66.08, 81.93)	<0.0001	100.00 (93.51, 100.00)	90.20 (78.59, 96.74)	0.0548
	Seropositive (≧1:8) rate % (95%CI)	96.08 (86.54, 99.52)	42.86 (30.46, 55.95)	<0.0001	95.74 (90.97, 98.42)	61.11 (52.02, 69.66)	<0.0001	98.18 (90.28, 99.95)	84.31 (71.41, 92.98)	0.0271
	GMT (1:) (95%CI)	23.09 (18.83, 28.32)	4.82 (3.77, 6.18)	<0.0001	48.36 (38.94, 60.07)	7.66 (6.34, 9.25)	<0.0001	66.47 (47.26, 93.48)	16.00 (11.41, 22.43)	<0.0001
**5 years after vaccination**	Seropositive (≧1:4) rate % (95%CI)	100.00 (93.02, 100.00)	77.78% (65.54, 87.28)	0.0003	100.00 (97.42, 100.00)	82.54 (74.77, 88.72)	<0.0001	100.00 (93.51, 100.00)	80.39 (66.88, 90.18)	0.0004
	Seropositive (≧1:8) rate % (95%CI)	88.24 (76.13, 95.56)	60.32% (47.20, 72.43)	0.0009	87.94 (81.40, 92.82)	65.08 (56.08, 73.35)	<0.0001	92.73 (82.41, 97.98)	66.67 (52.08, 79.24)	0.0008
	GMT (1:) (95%CI)	10.79 (9.01, 12.91)	6.93 (5.55, 8.66)	0.0025	13.60 (11.81, 15.68)	7.61 (6.47, 8.95)	<0.0001	17.26 (13.71, 21.72)	9.04 (6.71, 12.19)	0.0008
**8 years after vaccination**	N	47	60		128	106		43	38	
	Seropositive (≧1:4) rate % (95%CI)	100 (92.45, 100.00)	86.67 (75.41, 94.06)	0.0088	99.22 (95.72, 99.98)	92.45 (85.67, 96.69)	0.0123	100 (91.78, 100.00)	92.11 (78.62, 98.34)	0.0989
	Seropositive (≧1:8) rate % (95%CI)	87.23 (74.26, 95.17)	63.33 (49.90, 75.41)	0.0052	86.72 (79.59, 92.07)	76.42 (67.18, 84.12)	0.0409	95.35 (84.19, 99.43)	86.84 (71.91, 95.59)	0.2440
	GMT (1:) (95%CI)	11.74 (8.84, 15.59)	8.48 (6.60, 10.88)	0.0866	13.17 (11.29, 15.35)	9.80 (8.30, 11.56)	0.0103	17.07 (13.29, 21.91)	13.09 (9.80, 17.49)	0.1628

Abbreviations: IPS, 5-year immune persistence set; IPS2, 8-year immune persistence set; CI, confidence interval; GMT, geometric mean titer; VarV, varicella vaccine.

## Data Availability

The data used to support the findings of this study are included within the Supplementary Information files. The code is not available for commercial use but can be made available for research use upon reasonable request.

## Supplementary Materials

The following supporting information can be downloaded at: https://www.mdpi.com/article/10.3390/vaccines13101024/s1, Table S1: Demographics of participants included/not included in the persistent analysis; Table S2: Seropositive rates and GMTs measured in different study sites.
