# Correction: Wang et al. Immune Persistence Following a Single Dose of Varicella Vaccine: 5-Year and 8-Year Follow-Up of a Phase 3, Randomized, Double-Blind, Placebo-Controlled Trial. *Vaccines* 2025, *13*, 1024

**DOI:** 10.3390/vaccines14010095

**Published:** 2026-01-20

**Authors:** Yanxia Wang, Xiangling Lei, Lili Huang, Yuehong Ma, Hongxue Yuan, Dongyang Zhao, Fanhong Meng

**Affiliations:** 1 Henan Centers for Disease Control and Prevention, Zhengzhou 450046, China; wangyanxia99@163.com (Y.W.); 13643826177@163.com (L.H.); 2 Sinovac Holding Group Co., Ltd., Beijing 100085, China; leixiangling9199@sinovac.com (X.L.); mayuehong8757@sinovac.com (Y.M.); yuanhx2052@sinovac.com (H.Y.); 3 Sinovac (Dalian) Vaccine Technology Co., Ltd., Dalian 116620, China

There was an error in the original publication [1]. In the Materials and Methods, Section 2.3 “Vaccines”, the potency of the varicella vaccine (VarV) was mistakenly reported as “approximately 2.2 lgPFU per dose (0.5 mL)”, which does not reflect the release specification of the investigational product used in this study. Therefore, the sentence “The potency of the VarV was approximately 2.2 lgPFU per dose (0.5 mL)” should be corrected to “The potency of the VarV was not less than 3.3 lgPFU per dose (0.5 mL).”

The authors state that the scientific conclusions are unaffected. This correction was approved by the Academic Editor. The original publication has also been updated.
