# Supplementary material for: Immune Persistence Following a Single Dose of Varicella Vaccine: 5-Year and 8-Year Follow-Up of a Phase 3, Randomized, Double-Blind, Placebo-Controlled Trial
Source: Vaccines (Basel). 2025 Sep 22;13(10):1024. doi: 10.3390/vaccines13101024 (PMC12567696; doi:10.3390/vaccines13101024)
Supplement: Supplementary file 1 [file vaccines-13-01024-s001.zip › vaccines-3834651-supplementary.pdf]

Supplementary Table S1. Demographics of participants included/not included in the persistent analysis

Variable	Included in the persistent analysis	Not included in the persistent analysis	*p*-value
**N**	487	216	
**Age of vaccination (years, mean ± SD)**	6.3±2.77	6.8±3.36	0.0882
**Gender (male, %)**	50.1%	56.9%	0.0938
**GMT before vaccination (1:) (95% CI)**	7.03 (6.38, 7.75)	7.75 (6.56, 9.16)	0.3216
**GMT 30 days after vaccination (1:) (95% CI)**	19.03 (16.69, 21.71)	21.02 (17.09, 25.85)	0.4185
**Seropositive (≧1:4) rate before vaccination % (95%CI)**	72.07 (67.86, 76.02)	68.06 (61.39, 74.22)	0.2795
**Seropositive (≧1:4) rate before vaccination % (95%CI)**	55.03 (50.49, 59.51)	56.94 (50.05, 63.64)	0.6375
**Seropositive (≧1:8) rate before vaccination % (95%CI)**	86.24 (82.86, 89.18)	84.26 (78.70, 88.85)	0.4892
**Seropositive (≧1:8) rate before vaccination % (95%CI)**	79.06 (75.17, 82.59)	80.56 (74.64, 85.61)	0.6493

Supplementary Table S2. Seropositive rates and GMTs measured in different study sites

Study site	Variable	VarV group	Placebo group	*p*-value
**Biyang**				
	GMT before vaccination (1:) (95% CI)	5.77 (4.82,6.91)	6.50 (5.37,7.86)	0.7912
	GMT 30 days after vaccination (1:) (95% CI)	33.66 (27.31, 41.49)	7.21 (5.90, 8.81)	<0.0001
	GMT 5 years after vaccination (1:) (95% CI)	13.51 (11.45,15.95)	10.62 (9.05,12.45)	0.0392
	GMT 8 years after vaccination (1:) (95% CI)	14.40 (12.06,17.19)	11.09 (9.38,13.10)	0.0348
	Seropositive (≧1:4) rate before vaccination % (95%CI)	43.90 (34.97,53.13)	53.33 (44.01,62.49)	0.1414
	Seropositive (≧1:4) rate 30 days after vaccination % (95%CI)	100 (97.05,100.00)	71.67 (62.72,79.51)	<0.0001
	Seropositive (≧1:4) rate 5 years after vaccination % (95%CI)	100 (97.05,100.00)	90.83 (62.72,79.51)	<0.0001
	Seropositive (≧1:4) rate 8 years after vaccination % (95%CI)	100 (96.55,100.00)	91.53 (84.21,95.97)	0.0016
	Seropositive (≧1:8) rate before vaccination % (95%CI)	43.90 (34.97,53.13)	53.33 (44.01,62.49)	0.4892
	Seropositive (≧1:8) rate 30 days after vaccination % (95%CI)	94.31 (88.63,97.68)	58.33 (48.98,67.26)	<0.0001
	Seropositive (≧1:8) rate 5 years after vaccination % (95%CI)	85.37 (77.86,91.09)	80.83 (72.64,87.44)	0.3456
	Seropositive (≧1:8) rate 8 years after vaccination % (95%CI)	91.43 (84.35,96.01)	81.73 (72.95,88.63)	0.0396
**Xiangcheng**				
	GMT before vaccination (1:) (95% CI)	7.96 (6.52,9.71)	8.19 (6.63,10.11)	0.8446
	GMT 30 days after vaccination (1:) (95% CI)	58.85 (47.06,73.60)	8.72 (7.03,10.82)	<0.0001
	GMT 5 years after vaccination (1:) (95% CI)	13.84 (12.20,15.69)	5.59 (4.76,6.57)	<0.0001
	GMT 8 years after vaccination (1:) (95% CI)	12.75 (10.85,14.99)	8.82 (7.32,10.62)	0.0032
	Seropositive (≧1:4) rate before vaccination % (95%CI)	75.00 (66.43,82.34)	71.67 (62.72,79.51)	0.5560
	Seropositive (≧1:4) rate 30 days after vaccination % (95%CI)	100 (97.07,100.00)	72.50 (63.60,80.25)	<0.0001
	Seropositive (≧1:4) rate 5 years after vaccination % (95%CI)	100 (97.07,100.00)	70.83 (61.84,78.77)	<0.0001
	Seropositive (≧1:4) rate 8 years after vaccination % (95%CI)	99.12 (95.17,99.98)	90.00 (82.38,95.10)	0.0027
	Seropositive (≧1:8) rate before vaccination % (95%CI)	59.68 (50.49,68.39)	63.33 (54.05,71.94)	0.5574
	Seropositive (≧1:8) rate 30 days after vaccination % (95%CI)	98.39 (94.30,99.80)	64.71 (54.90,72.71)	<0.0001
	Seropositive (≧1:8) rate 5 years after vaccination % (95%CI)	92.74 (86.67,96.63)	47.50 (38.31,56.82)	<0.0001

Seropositive (≧1:8) rate 8 years after vaccination % (95%CI)	85.84 (78.03,91.68)	67.00 (56.88,76.08)	0.0011
